# Effects of the STriatal Enriched Tyrosine Phosphatase (STEP) Inhibitor TC‐2153 on Hippocampal Long‐Term Depression and Synaptic Transmission: Paradoxical Effect on Phosphatase Activity and Role of Adenosine

**DOI:** 10.1002/cns.70843

**Published:** 2026-03-24

**Authors:** Valentina Chiodi, Rita Pepponi, Lucia Gaddini, Zaira Boussadia, Emilia Marchei, Manuela Pellegrini, Cinzia Mallozzi, Maria Rosaria Domenici

**Affiliations:** ^1^ National Centre for Drug Research and Evaluation Istituto Superiore di Sanità Rome Italy; ^2^ National Centre on Addiction and Doping Istituto Superiore di Sanità Rome Italy; ^3^ Department of Neuroscience Istituto Superiore di Sanità Rome Italy

**Keywords:** adenosine, long‐term depression, striatal enriched tyrosine phosphatase, synaptic transmission

## Abstract

**Aims:**

This study aimed to explore the effects of TC‐2153, the STriatal Enriched Tyrosine Phosphatase (STEP) inhibitor, on Long‐Term Depression (LTD) and basal synaptic transmission in hippocampal slices.

**Methods:**

Extracellular field potentials were recorded in the CA1 area of the hippocampal slices. LTD was induced by low‐frequency stimulation and by metabotropic glutamate receptor stimulation. The activity of STEP was measured in hippocampal slices and in SH‐SY5Y cell culture by a colorimetric assay using p‐nitrophenol as a substrate. To evaluate adenosine levels, adenosine was extracted from hippocampal slices homogenates and measured by HPLC.

**Results:**

TC‐2153 3 μM, applied to the slices one hour before and then along the electrophysiological recordings, blocked both forms of LTD. When hippocampal slices were treated with TC‐2153 for shorter periods, 10–20 min, TC‐2153 reduced synaptic transmission and increased STEP activity with an adenosine A1 receptor‐dependent mechanism. Consistently, we found that TC‐2153 increased adenosine levels in hippocampal slices. The increase in STEP activity after brief TC‐2153 treatment has been confirmed in SH‐SY5Y cells.

**Conclusion:**

Our study confirms the role of STEP in LTD and reveals a new mechanism of action for TC‐2153. The unexpected adenosine‐dependent activation of STEP by TC‐2153 has significant implications for both basic research and potential therapeutic applications.

## Graphical abstract

In hippocampal slices, treatment with TC‐2153 for 1–2 h blocks the two forms of long‐term depression, mGlu‐LTD and NMDA‐LTD, by inhibiting the STriatal Enriched tyrosine Phosphatase (STEP). On the contrary, short‐term TC‐2153 application (10–20 min) increases STEP activity through a mechanism involving adenosine release and adenosine A1 receptor stimulation, which results in a reduction of basal synaptic transmission.

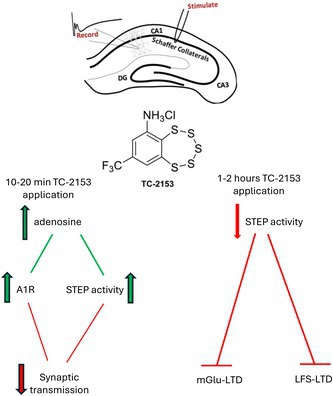

## 1 Introduction

Activity‐dependent changes in synaptic strength such as those occurring during long‐term potentiation (LTP) or long‐term depression (LTD) are considered crucial to understand the cellular and molecular mechanisms of learning and information storage in the brain. LTP and LTD, the two major forms of synaptic plasticity in the brain, have been extensively studied in different brain areas, and in particular in the hippocampus. Both forms of synaptic plasticity are developmentally regulated, with LTD that prevails in neonatal and juvenile animals, while LTP is more easily inducible in adult animals [1, 2, 3, 4]. Here, we focused on the two principal forms of hippocampal LTD, the metabotropic glutamate receptor (mGluR)‐dependent LTD and NMDAR‐dependent LTD, and we investigated the involvement of the STriatal Enriched Tyrosine Phosphatase (STEP), a brain‐specific tyrosine phosphatase implicated in the pathophysiology of several neuropsychiatric diseases [5].

In the CA1 area of hippocampal slices mGluR‐dependent LTD (mGlu‐LTD) is induced by the application of the group I mGluR agonist, typically 3,5‐dihydroxyphenylglycine (DHPG). Several signaling pathways responsible for mGluR‐LTD have been identified, showing the involvement of multiple mechanisms [6, 7]. Among them, a prominent role seems to be played by protein tyrosine phosphatases, which dephosphorylate AMPA receptor subunits inducing their internalization and removal from synapses [6, 8, 9].

NMDA receptor‐dependent LTD is usually induced in the CA1 area of the hippocampus by low‐frequency stimulation (LFS) of the Schaffer collaterals (LFS‐LTD). This form of LTD is NMDA receptor dependent, requires an increase in postsynaptic calcium, activation of serine/threonine phosphatases, dephosphorylation of the AMPA receptor subunits, and internalization of the receptors [9, 10, 11]. Thus, protein phosphatase activation and AMPA receptor internalization are common steps in mGlu‐LTD and LFS‐LTD.

STEP is a tyrosine phosphatase widely expressed in the brain where it regulates neuronal survival and synaptic functions through tyrosine dephosphorylation of its substrates, which include NMDA and AMPA receptors subunits, the Src non‐receptor protein tyrosine kinase Fyn, the focal adhesion kinase proline‐rich tyrosine kinase 2 (Pyk2), and two members of the mitogen activated protein kinase family, ERK1/2 and p38 [12, 13, 14, 15, 16, 17, 18, 19]. Considering the role played by STEP substrates in the regulation of synaptic activity, together with the observation that glutamate receptor endocytosis is regulated by STEP‐mediated tyrosine dephosphorylation [20, 21, 22, 23], a role of STEP in the modulation of synaptic plasticity is not surprising. Indeed, STEP is implicated in the induction of LTP in different brain areas [15, 24, 25, 26] and some studies demonstrated a role for STEP in specific forms of LTD. In particular, activation of STEP is involved in Aβ‐enhanced LTD in the dentate gyrus of the hippocampus [27] and in the exaggerated mGluR‐LTD in the hippocampal CA1 area of a mouse model of Fragile X syndrome [28]. Recently, mGluR‐LTD in CA2 area of the hippocampus was found to be STEP‐dependent [29]. However, as far as we know, no study has investigated the direct involvement of STEP in LFS‐LTD in the CA1 area of the hippocampus. In the present study, we investigated the role of STEP in hippocampal LTD by using the STEP inhibitor TC‐2153 [30]. We evaluated TC‐2153 effects both on mGlu‐LTD and LFS‐LTD at the Schaffer collaterals‐CA1 synapse in mice hippocampal slices, to verify whether STEP could represent a common mechanism in the two forms of hippocampal LTD. In addition, we assessed the effect of TC‐2153 on hippocampal basal synaptic transmission. The results demonstrate that STEP activation is necessary in order to induce both forms of LTD. Moreover, acute and brief application of TC‐2153 induces (i) reduction of synaptic transmission mediated by adenosine A1 receptor (A1R) activation; (ii) release of adenosine in hippocampal slices; and (iii) an unexpected increase of STEP activity in both hippocampal slices and in SH‐SY5Y cell culture. Our study confirms the role of STEP in LTD and reveals a new mechanism of action for TC‐2153 involving adenosine.

## 2 Methods

### 2.1 Drugs

(S)‐3,5 Dihydroxyphenylglycine (DHPG), N6‐cyclopentyladenosine (CPA), and DPCPX are from Tocris (Bio‐Techne, Milan, Italy); TC‐2153 is from Sigma‐Aldrich (St. Louis, MO, USA). The polyclonal antibodies anti‐STEP are from Cell Signaling Technology (Danvers, MA, USA). Except for DHPG that was dissolved in H_2_O, all the other drugs were dissolved in DMSO, and the maximum final percentage in all the treatments did not exceed 0.01%. Protein A Trysacryl beads are from Thermo Fisher Scientific (Waltham, Massachusetts, USA), and para‐nitrophenyl phosphate (p‐NPP) is from Sigma‐Aldrich.

### 2.2 Animals

C57Bl/6 mice were used. The animals were kept in standard cages under controlled temperature (22°C), humidity (55%), and lighting conditions (on a 12 h light/dark cycle), with water and food ad libitum. Animal procedures were carried out according to the principles outlined in the European Community Guidelines for Animal Care, DL 26/2014, via the application of the European Communities Council Directive, 2010/63/EU, and the FELASA and ARRIVE guidelines. All animal procedures were approved by the Italian Ministry of Health (D9997.N.TNL). We used both male and female mice. No randomization to allocate subjects in the study or blinding was performed.

### 2.3 Hippocampal Slice Electrophysiology

Hippocampal slices were prepared as previously described [31]. Briefly, animals were decapitated, the brains removed and placed in ice‐cold artificial cerebrospinal fluid (ACSF) containing the following (in mM): 126 NaCl, 3.5 KCl, 1.2 NaH_2_PO_4_, 1.2 MgCl_2_, 2 CaCl_2_, 25 NaHCO_3_, and 11 glucose, pH 7.3, saturated with 95% O_2_ and 5% CO_2_. The brains were sectioned by using a vibratome (5100 mz, Campden Instruments) to obtain parasagittal slices (400 μm) containing the hippocampus. After incubation in a holding chamber with ACSF (22°C–25°C) for at least 60 min, the slices were placed in a submerged recording chamber and superfused with ACSF at a flow rate of 2.5–3 mL/min (32°C–33°C). Field excitatory post‐synaptic potentials (fEPSPs) were recorded in the stratum radiatum of CA1 area of the hippocampus with a glass microelectrode filled with 2 M NaCl (1–3 MΩ resistance) after stimulation of the Schaffer collaterals through an insulated bipolar twisted NiCr electrode and an isolated stimulator (Isostim A320, WPI Instruments). Traces were acquired, amplified and analyzed with DAM‐80 AC differential amplifier (WPI Instruments) and with the WinLTP software (WinLTP Ltd). Stimuli (100 μsec duration, delivered every 20 s) were set to an intensity that evoked half‐maximal fEPSPs, and three consecutive fEPSPs were averaged. LFS‐LTD was induced by a stimulation protocol consisting of 1200 stimuli delivered at 2 Hz. mGlu‐LTD was induced by 10 min bath application of 100 μM DHPG. Synaptic transmission was recorded for 50 min after the induction of LTD and 10 min of stable baseline recordings preceded LFS or drug application. Changes in synaptic transmission were measured as changes in fEPSP slope and expressed as percentage of the average slope measured during the 10 min that preceded LFS or DHPG application. To evaluate paired pulse facilitation (PPF), an index of pre‐synaptic neurotransmitter release, we measured the ratio between the slope of the second (R2) and the first response (R1) evoked by 2 consecutive stimuli, 50 ms apart.

### 2.4 Hippocampal Slice Treatment

To evaluate drug effects on STEP activity and on adenosine levels, we used hippocampal slices prepared from the hippocampus of adult mice cut using a tissue chopper. Slices were stored in oxygenated ACSF for at least one hour and then assigned to the different group treatments.

### 2.5 SH‐SY5Y Cell Culture and Treatment

SH‐SY5Y human neuroblastoma cells were grown in Dulbecco's modified Eagle's medium plus F12 in a 1:1 ratio, containing 10% bovine serum, 1% L‐glutamine, and 1% penicillin/streptomycin (Euroclone, Italy), at 37°C in a humidified 5% CO_2_ atmosphere and used within passage 30. Cells were seeded in Petri dishes at a density of 2.5 × 10^6^/10 mL/petri. Treatments were performed 24 h after the onset of the culture.

### 2.6 STEP Activity

STEP activity was measured in hippocampal slices and in SH‐SY5Y cells. Hippocampal slices (4–5 slices for each experimental point) were homogenized in ice‐cold RIPA buffer, 1 mM PMSF, and complete protease inhibitors cocktail, kept for 1 h in ice, and centrifuged at 10,000 g for 10 min. SH‐SY5Y cells were lysed in ice‐cold RIPA buffer and followed the same procedure as hippocampal slices. After centrifugation, supernatants (1 mg/mL) from hippocampal slices and SH‐SY5Y cells were incubated with 50% (w/v) Protein A Trysacryl beads for 2 h at 4°C. The supernatants were incubated overnight at 4°C in a rotating wheel with a polyclonal anti‐STEP antibody (1–2 μg/sample, Cell Signaling Technology, Danvers, MA, USA). The immunocomplexes were precipitated by the addition of 50% (w/v) Protein A Trysacryl beads. To measure the activity of STEP, the immunoprecipitate complexes were suspended, after extensive washing, in 150 μL of assay buffer (in mM: 25 Hepes, pH 7.4, 20 MgCl_2_, 0.1 PMSF) containing 15 mM p‐NPP and incubated for 60 min at 37°C under gentle stirring. Phosphatase activity of STEP was measured in the supernatants by colorimetric quantitation of the formation of p‐nitrophenol at 410 nm using a spectrophotometer.

### 2.7 Western Blotting Analysis

Hippocampal slices were homogenized in RIPA buffer as described above and analyzed by Western Blotting (WB) analysis according to Mallozzi et al. (2023) [32]. The presence of STEP61 and STEP46 was revealed on nitrocellulose using the polyclonal anti‐STEP antibody. The immunoreactive bands were detected via chemiluminescence coupled to peroxidase activity (ECL kit; Euroclone, Milano, Italy).

### 2.8 Measurement of Adenosine Levels in Hippocampal Slices

After a thorough homogenization in acetonitrile solution (1 mg/10 μL) with hydrochloric acid (0.83%), adenosine was extracted from hippocampal slice by sonication for 60 s. The homogenates of hippocampal tissue were centrifuged at 12,000 rpm for 10 min at 4°C. Supernatant was solubilized with 100 μL of 0.1% formic acid and methanol (90:10, v/v). Ten μl were injected for quantification of adenosine. Analysis was performed with a ultra‐high performance liquid chromatography system (Waters Acquity UPLC, Waters Corporation, Milford, MA, USA) coupled with a triple quadrupole mass spectrometer (Waters Xevo TQ, Waters Corporation), following the protocol by Bergh et al. (2016) [33]. Chromatography was carried out using the Acquity HSS T3 column (Waters) and a linear gradient elution with 0.1% formic acid (mobile phase A) and methanol (mobile phase B). The flow rate was kept constant at 0.4 mL/min. MS was performed with Electrospray ionization in a positive mode under the following conditions: voltage = 0.6 kV, desolvation T = 450°C, source T = 150°C, cone gas flow rate = 60 L/h, desolvation gas flow rate = 1000 L/h, collision gas flow rate = 0.11 mL/min.

### 2.9 Statistical Analysis

The statistical analysis was performed with GraphPad Prism software version 6.07 (San Diego, CA, USA). Results are expressed as mean values ± standard error of the mean (SEM) and analyzed with the Student *t*‐test or Mann–Whitney *U*‐test when the difference between two groups is considered. When more groups are evaluated, one‐way ANOVA or Kruskal‐Wallis test followed by Dunn's multiple comparison test were used. Significance was accepted at *p* ≤ 0.05.

## 3 Results

### 3.1 TC‐2153 Prevents LTD in Hippocampal Slices

**FIGURE 1:**
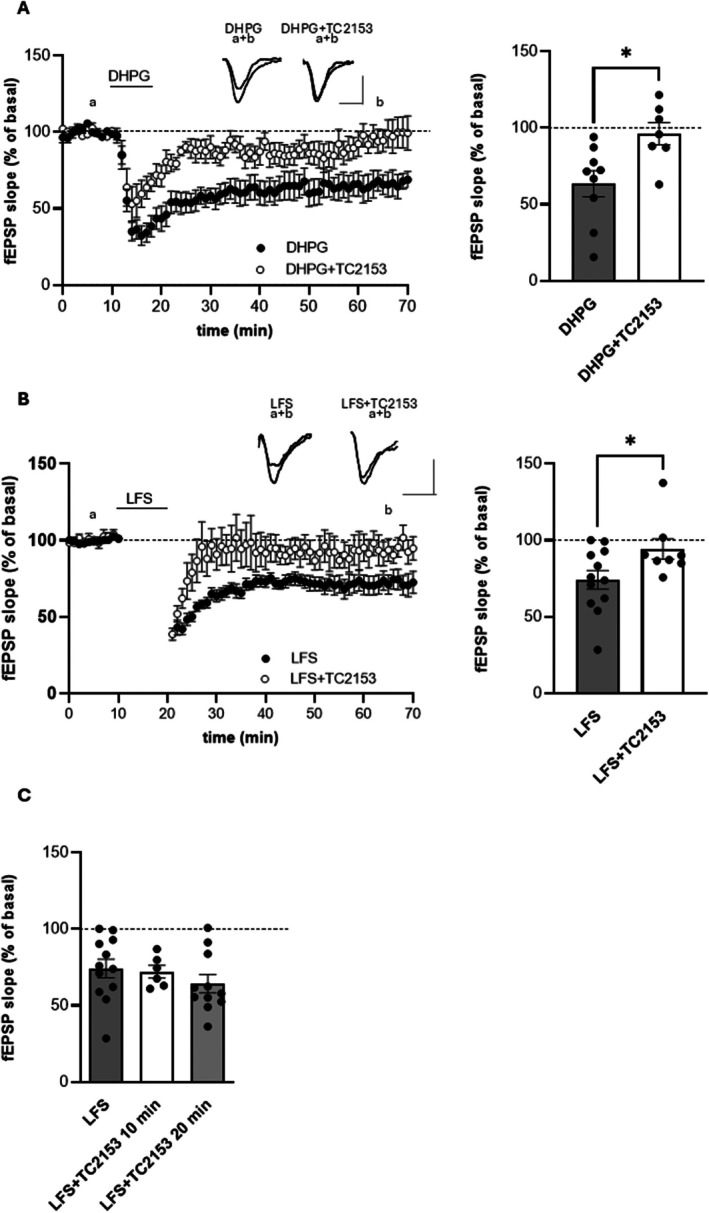
TC‐2153 prevents LTD in the hippocampus. (A) Time courses of fEPSP slope: 10 min slice perfusion with DHPG 100 μM induced mGlu‐LTD that was prevented by slice pretreatment with 3 μM TC‐2153. Insets show fEPSPs recorded in basal condition (a) and 50 min after DHPG wash‐out (b); calibration: 0.5 mV, 10 ms. The bar graph on the right shows a significant difference in the percentage of fEPSP slope 50 min after DHPG washout between DHPG and DHPG+TC‐2153 (*p < 0.05, Student *t*‐test). (B) Time courses of fEPSP slope: LFS (1200 stimuli at 2 Hz) induced LTD that was prevented by slice pretreatment with 3 μM TC‐2153. Insets show fEPSPs recorded in basal condition (a) and 50 min after LFS (b). The bar graph on the right shows a significant difference in the fEPSP slope between LFS and LFS + TC‐2153 (*p < 0.05, Student *t*‐test) measured 50 min after LFS. (C) Short application of TC‐2153 (10 and 20 min) did not change LTD induction.

**FIGURE 2:**
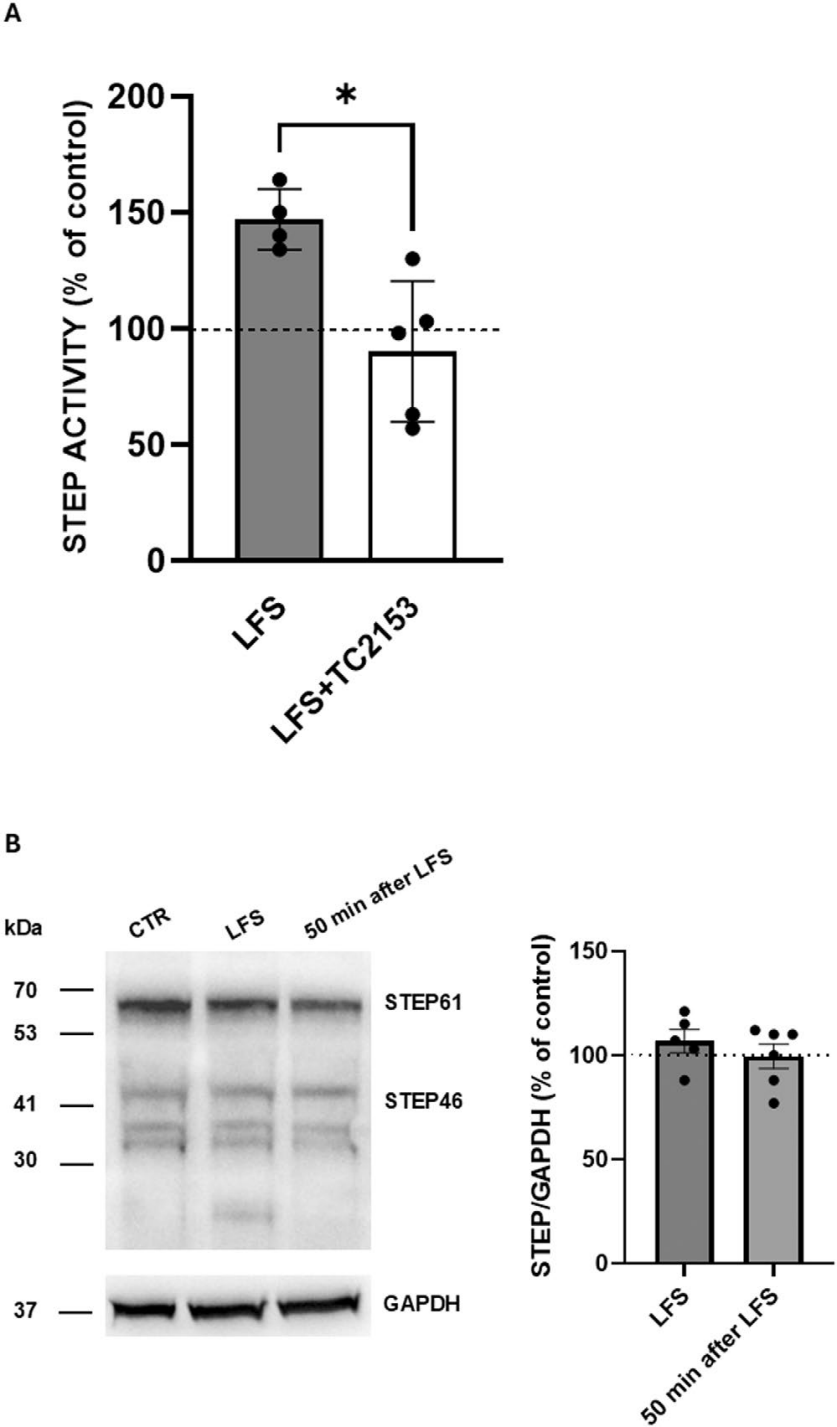
LFS increases STEP activity with no changes in STEP protein levels. (A) LFS increases STEP activity, an effect that was prevented by slice pretreatment with TC‐2153 (*p < 0.05, Mann–Whitney test). (B) WB experiment and densitometric analysis with STEP antibody performed in hippocampal slices soon after LFS (*n* = 5) and 50 min after the end of LFS (*n* = 6). Each point represents a pool of 4 hippocampal slices. Equal amounts of proteins were transferred to nitrocellulose membrane for WB. The presence of STEP46 and STEP61 was revealed via the polyclonal antibody and the bands were detected via chemiluminescence peroxidase activity (ECL). The molecular mass markers in kDa are indicated on the left. As a loading control, GAPDH levels were evaluated.

In hippocampal slices from 2‐week‐old mice, we measured the ability of the mGluR agonist to induce LTD. Ten minutes of slice perfusion with DHPG 100 μM induced long‐ lasting synaptic depression of fEPSP (63.45% ± 8.55% of baseline 50 min after DHPG washout, *n* = 9) as shown in Figure 1A. When slices were treated with 3 μM TC‐2153 for at least one hour before and then during fEPSP recordings, DHPG did not elicit anymore LTD (96.24% ± 7.3% of baseline, *n* = 7, *p* < 0.05 with respect to DHPG alone, Student *t*‐test Figure 1A), demonstrating a role of STEP in mGlu‐LTD. We then evaluated NMDA receptor‐dependent LTD induced by LFS (LFS‐LTD). fEPSPs were evoked in the CA1 area after stimulation of the Schaffer collaterals. After 10 min of baseline recording LTD was induced by LFS of the afferents, consisting in 1200 stimuli at 2 Hz. This stimulation protocol induced a reduction of fEPSP measured 50 min after LFS (74.23 ± 6.08 of baseline values, *n* = 12, Figure 1B). When we incubated hippocampal slices with the STEP inhibitor TC‐2153 (3 μM), one hour before and then during fEPSP recordings, LFS failed to induce LTD (102.5 ± 12.9 of baseline values, *n* = 10, *p* < 0.05 with respect to LFS alone, Student *t*‐test, Figure 1B). Shorter duration of TC‐2153 application (10 and 20 min) did not modify the induction of LFS‐LTD with respect to LFS alone (72.18 ± 4.1 of baseline values, *n* = 6, after 10 min TC‐2153 application and 64.2 ± 5.9 of baseline values, *n* = 11, after 20 min TC‐2153 application, *p* > 0.05 one‐way ANOVA, Figure 1C). To demonstrate that TC‐2153 exerted its effect by specifically inhibiting STEP, we first verified whether the LFS modulated STEP activity. To this end, we measured STEP activity in hippocampal slices in control conditions (i.e., after 10 min of baseline recordings) and soon after LFS. We found that LFS induced an increase in STEP activity (147% ± 6.55% of control values, *n* = 4), an effect abolished by slice pre‐treatment, for at least one hour and then along the electrophysiological recordings, with 3 μM TC‐2153 (90.2% ± 13.5% of control values, *n* = 5, *p* < 0.05 vs LFS alone, Mann–Whitney *U*‐test, Figure 2A). Thus, an increase in STEP activity is necessary for LFS‐LTD to occur. Then we asked whether the induction of LTD could result in changes in STEP protein levels. By WB experiments, we evaluated STEP protein levels in control condition, immediately after LFS, and 50 min after LFS. As shown in Figure 2B, we found no changes in protein levels for both the isoforms STEP46 and STEP61. The graph bar shows the STEP46/61 level normalized with GAPDH. Interestingly, although STEP46 was reported to be absent in the hippocampus [34], we could detect this isoform also in this area, in agreement with the study by Moskaliuk et al. [35].

### 3.2 TC‐2153 Induces Reduction of Basal Synaptic Transmission Through Adenosine A1 Receptor Activation

**FIGURE 3:**
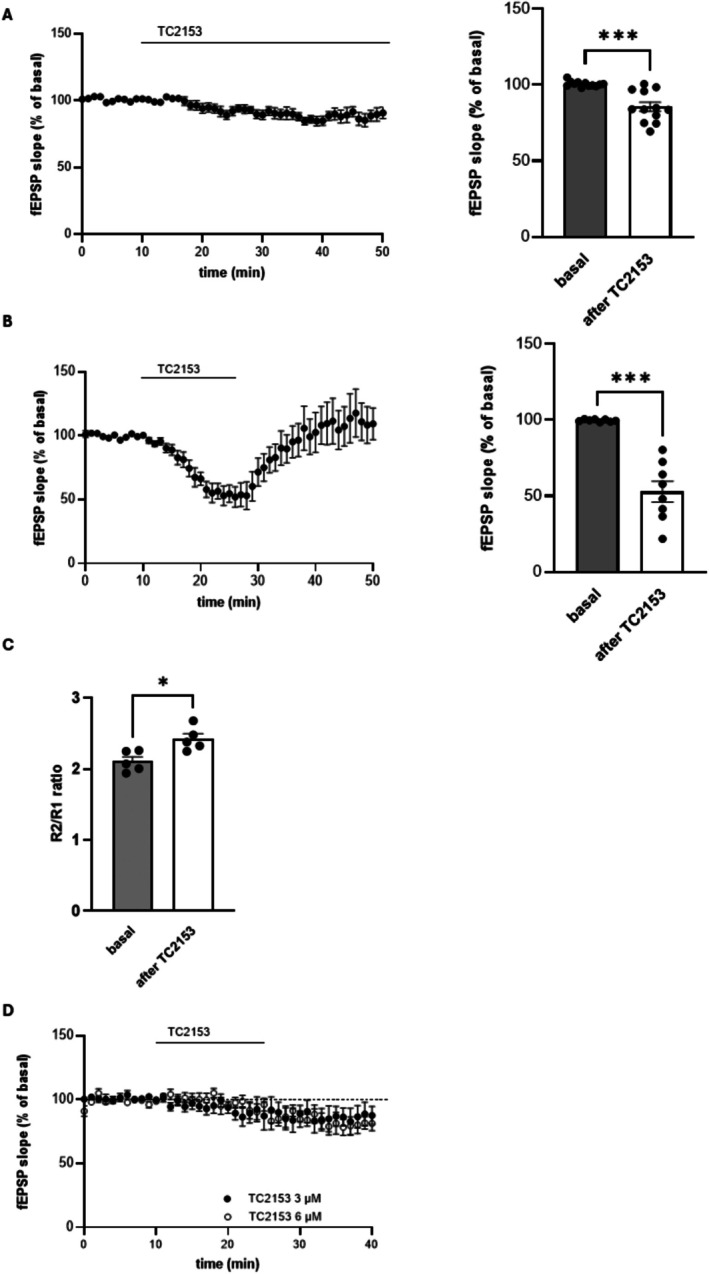
TC‐2153 reduces basal synaptic transmission in hippocampal slices. (A) Acute TC‐2153 application (3 μM) significantly reduces synaptic transmission in hippocampal slices from neonatal mice (*p < 0.001, paired *t*‐test). (B) In adult mice, acute perfusion of hippocampal slices with 3 μM TC‐2153 induced a strong reduction of synaptic transmission (*p < 0.001, paired *t*‐test). (C) TC‐2153 induces an increase of R2/R1 ratio in hippocampal slices of adult mice (*p < 0.05, Student *t*‐test). (D) In slices from neonatal mice TC‐2153 3 μM and 6 μM has a similar effect on basal synaptic transmission.

For LTD experiments, hippocampal slices have been treated with TC‐2153 3 μ 60 min before and then along the electrophysiological recordings, that is, for a total of almost 2 h. However, nothing is known about the acute effect of TC‐2153 on basal synaptic transmission. To this end, fEPSPs were recorded in CA1 area of hippocampal slices of neonatal mice (2 weeks old). When a stable baseline was obtained, slices were perfused with 3 μM TC‐2153. TC‐2153 induced a reduction of fEPSP measured after 40 min of application (85.55% ± 2.97% of basal values, *n* = 12, *p* < 0.001 paired *t*‐test, Figure 3A). To confirm this effect of TC‐2153, we repeated the experiments in hippocampal slices from adult mice (3 months old). We found that the effect of 3 μM TC‐2153 was much stronger than in neonatal mice, and after only 15 min of application, we observed a reduction of fEPSPs that reached 52.83% ± 6.9% of baseline values (*n* = 8, *p* < 0.001, paired Student *t*‐test) and recovered upon the washout (Figure 3B). The reduction of fEPSP was associated to an increase in PPF (R2/R1 ratio: 2.1 ± 0.06 and 2.4 ± 0.07 in basal condition and after TC‐2153, respectively, *p* < 0.05, Student *t*‐test, *n* = 5, Figure 3C), suggesting a reduction in neurotransmitter release. In neonatal slices we increased TC‐2153 concentration up to 6 μM and we found a similar effect to those obtained with 3 μM (80.4 ± 5.7, *n* = 5 and 86.5 ± 7.9, *n* = 6, respectively, Figure 3D).

### 3.3 TC‐2153 Induces Adenosine Release in Hippocampal Slices

**FIGURE 4:**
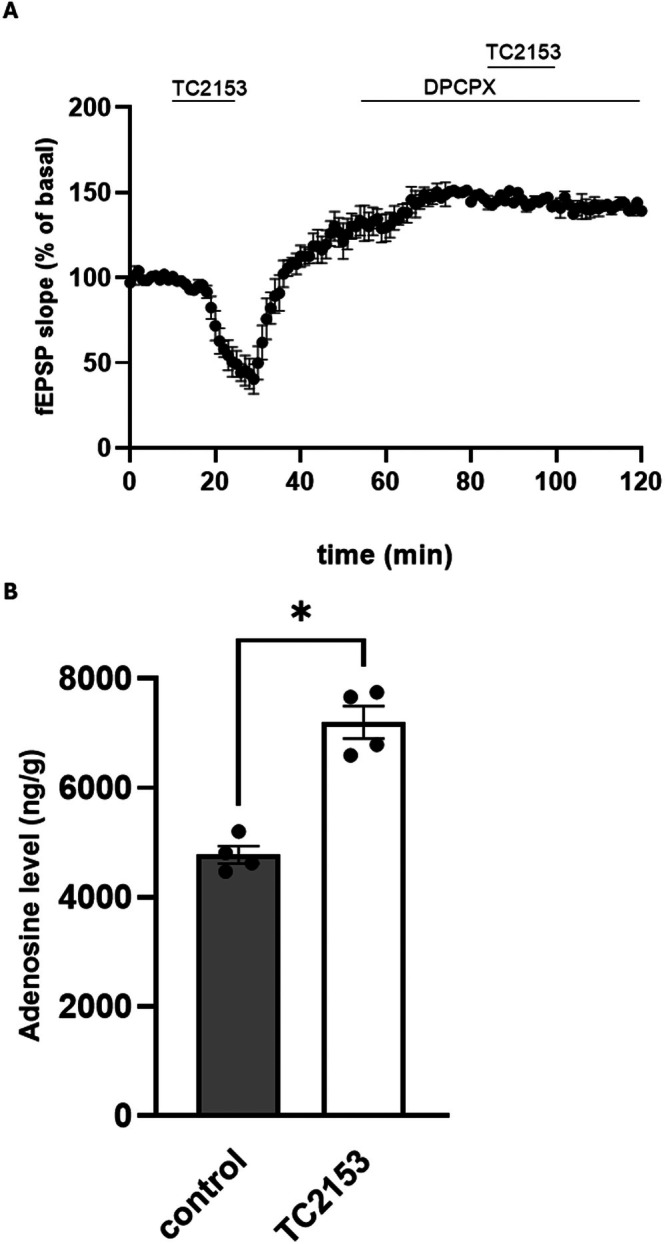
TC‐2153‐induced reduction of synaptic transmission is dependent on A1R stimulation. (A) The selective A1R antagonist DPCPX applied 30 min before and then along with TC‐2153 prevents TC‐2153‐induced reduction of synaptic transmission. (B) Ten minutes application of 3 μM TC‐2153 to hippocampal slices induces a significant increase in adenosine levels (*p < 0.05, Mann–Whitney *U*‐test).

Since the effect of TC‐2153 is reminiscent of that produced in condition of adenosine release and A1R stimulation, we verified whether the effect of TC‐2153 on synaptic transmission could be mediated by A1R stimulation. We found that the selective A1R antagonist DPCPX 500 nM, applied 30 min before and then together with TC‐2153, prevented the reduction of the synaptic transmission (*n* = 3, Figure 4A), demonstrating the involvement of A1R in TC‐2153‐mediated effects.

Given these results, we investigated the possibility that TC‐2153 could increase adenosine levels. To this end, adult hippocampal slices were treated with TC‐2153 3 μM for 10 min and adenosine levels measured through HPLC. We found a significant increase in adenosine levels in slices incubated with TC‐2153 (control: 4775 ± 159.4 ng/g, *n* = 4; TC‐2153: 7199 ± 295.1 ng/g, *n* = 4, *p* < 0.05 Mann–Whitney *U*‐test, Figure 4B).

### 3.4 Time Course of TC‐2153 Effects on STEP Activity

**FIGURE 5:**
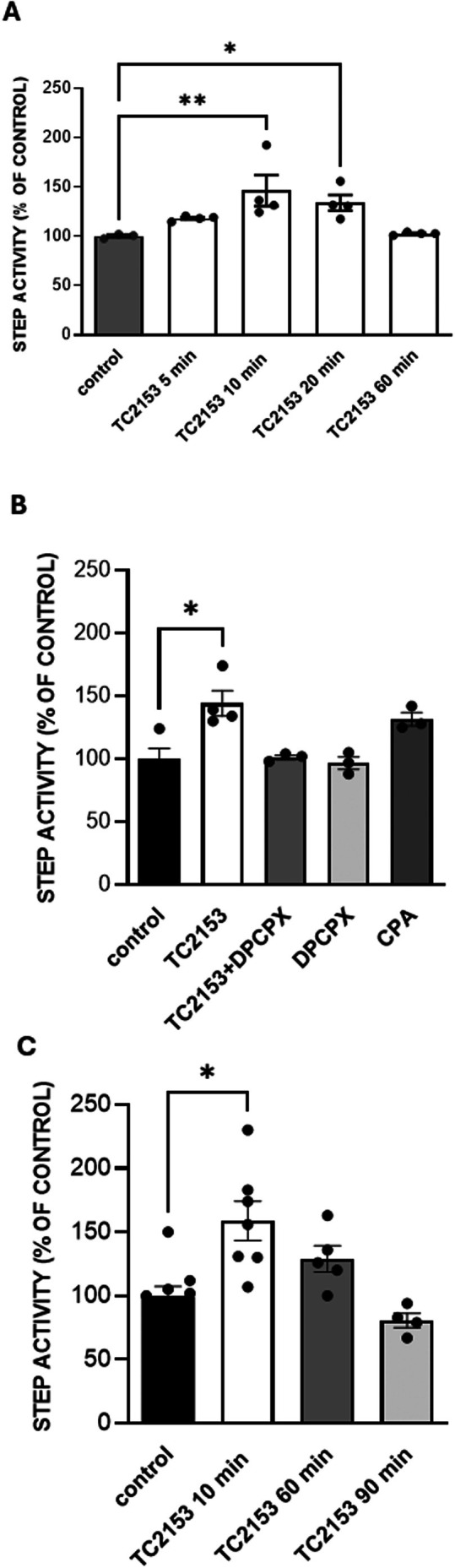
STEP activity in hippocampal slices and in SH‐SY5Y cells. (A) STEP activity in homogenates prepared from hippocampal slices treated with TC‐2153 3 μM was measured at different time points. STEP was immunoprecipitated by a specific polyclonal antibody from solubilized hippocampal slices samples containing the same protein content (each sample was obtained by pulling four hippocampal slices). The phosphatase activity of STEP was measured in the immunocomplex and expressed as percentage (control 100%) of the absorbance at 410 nm. The bar graphs represent the means ± SEM. *p < 0.05 and **p < 0.01 with respect to control (Kruskal‐Wallis test followed by Dunn's multiple comparison test). (B) STEP activity in homogenates prepared from hippocampal slices and measured as described above. The increase in STEP activity after 10 min application of TC‐2153 was mediated by A1Rs since it was prevented by DPCPX. CPA induces an increase, even though not statistically significant, of STEP activity. The bar graphs represent the means ± SEM. *p < 0.05 significantly different from control (Kruskal‐Wallis test followed by Dunn's multiple comparison test). (C) Time‐course of STEP activity, measured as described above, in lysates prepared from SH‐SY5Y neuroblastoma cells treated with 3 μM TC‐2153. The bar graphs represent the means ± SEM. *p < 0.05 significantly different from control (Kruskal‐Wallis test followed by Dunn's multiple comparison test).

The observation that TC‐2153 induced a reduction of synaptic transmission was surprising since the expected effect, if any, was an increase in synaptic transmission given the role of STEP in dephosphorylating glutamate receptors and promoting their removal from the synapses [22]. This, together with the result on adenosine release, raised doubts about the real action of TC‐2153 on STEP and prompted us to verify the effect of acute application of TC‐2153 on STEP activity. We measured STEP activity in homogenates prepared from hippocampal slices treated with TC‐2153 3 μM at different time points (5, 10, 20, and 60 min of application). We found that 10 and 20 min of TC‐2153 significantly increased STEP activity (146% ± 15.62% and 133.8 ± 7.95 of control values, *p < 0.05 and **p < 0.01 vs control, respectively, Kruskal Wallis followed by Dunn's multiple comparison test), which slowly returned to control levels after 60 min of application (Figure 5A). The increase in STEP activity after 10 min TC‐2153 application was mediated by A1R since it was prevented by DPCPX, the selective A1R antagonist (*p < 0.05 vs control, Kruskal Wallis followed by Dunn's multiple comparison test, Figure 5B). Notably, CPA, the selective A1R agonist, induced an increase, even though not statistically significant, of STEP activity (131.7 ± 5.24 of control values, Figure 5B).

To confirm the effect of TC‐2153 on STEP activity, we verified its effect on a neuroblastoma cell line. SH‐SY5Y cells were treated with 3 μM TC‐2153 for 10, 60 and 90 min: a significant increase of STEP activity was measured after 10 min of TC‐2153 application (158.7% ± 15.57% of control, *n* = 7, *p* < 0.05 vs control values, *n* = 9, Kruskal Wallis followed by Dunn's multiple comparison test), which decayed to control levels within 60–90 min (Figure 5C).

## 4 Discussion

The scientific interest in the STEP inhibitor TC‐2153 has been significantly increasing in the last years due to the reported effects in various models of neuropsychiatric disorders, suggesting its potential use as a drug for the treatment of multiple neurological conditions [28, 36, 37, 38]. Given this, it is important to investigate its effects on synaptic transmission and synaptic plasticity, particularly in brain regions that are critically involved in learning and memory processes. In the hippocampus, it is known that slice treatment with TC‐2153 improves LTP [26] but nothing is known on TC‐2153's effects on LTD and on basal synaptic transmission. We found that (i) TC‐2153 blocks mGlu‐LTD and LFS‐LTD in hippocampal slices, consistent with its inhibitory effect on STEP activity; (ii) acute hippocampal slice perfusion with TC‐2153 reduces basal synaptic transmission through a mechanism dependent on adenosine A1R; (iii) brief, 10 min, TC‐2153 treatment increases adenosine release in hippocampal slices; (iv) brief, 10–20 min, TC‐2153 treatment induces an unexpected and paradoxical activation of STEP mediated by adenosine A1 receptor, which declined to control levels after 60 min of application. Thus, our results highlight interesting, unexpected and previously unreported effects of TC‐2153.

The first evidence of a possible involvement of STEP in the induction of mGlu‐LTD came from the study of Zhang and collaborators (2008) who demonstrated that DHPG induced STEP translation and AMPA receptor endocytosis in a STEP‐dependent way [20]. In addition, studies in fragile X model mice showed that excess STEP protein correlated with the exaggerated mGluR‐LTD in CA1 hippocampal area [18]. A role for STEP in LTD has been identified also in CA2 area of the hippocampus, where mGluR‐LTD is more pronounced compared to CA1 area, and where application of STEP could rescue impairments in mGluR‐LTD [29]. In the present study, by using the STEP inhibitor TC‐2153, we confirmed that mGlu‐LTD in CA1 area of hippocampal slices is STEP‐dependent and highlighted, for the first time, a direct role of STEP in NMDA‐LTD. In fact, we demonstrated that LFS in hippocampal slices induced an increase in STEP activity that is crucial for LTD since TC‐2153 inhibited this increase and prevented LTD. However, no changes in STEP46 or in STEP61 protein levels have been detected in the hippocampus after LFS and LTD and, in contrast to what reported in previous studies, we could detect also in the hippocampus the isoform STEP46, which was supposed to be absent in this area [39, 40]. These results further demonstrate a critical role of STEP in synaptic plasticity and based on literature data and our results, we can conclude that TC‐2153 has an opposite effect on the two principal forms of synaptic plasticity, promoting and preventing LTP and LTD, respectively.

We did LTD experiments in hippocampal slices that were pre‐incubated for at least one hour and then along the electrophysiological recordings with TC‐2153, in line with previous studies on the synaptic effects of TC‐2153 [26, 30, 36]. However, no study had investigated so far the acute effects of TC‐2153 on synaptic transmission. Our results demonstrate that TC‐2153 reduced hippocampal basal synaptic transmission both in neonatal and in adult mice. We began investigating TC‐2153 effects on synaptic transmission in hippocampal slices from neonatal mice since we evaluated hippocampal LTD, which is an age‐dependent form of synaptic plasticity robustly express up to 17 days of age [2]. To verify whether TC‐2153‐mediated reduction of synaptic transmission was a more general phenomenon and not specifically related to the neonatal phase, we moved to adult hippocampal slices and found a similar effect but of greater magnitude. Moreover, acute application of TC‐2153 increased PPF ratio, consistently with a reduction of glutamate release. Since TC‐2153 electrophysiological effects were similar to those of an adenosine A1 receptor agonist (i.e., it reduced fEPSP and increased PPF), we decided to evaluate adenosine levels in hippocampal slices. Indeed, we found that TC‐2153 increased adenosine levels and the synaptic effects of TC‐2153 were blocked by the A1R antagonist DPCPX. Interestingly, TC‐2153‐induced reduction of synaptic transmission was stronger in adult than in neonatal mice. This result is in line with the demonstration that in adult mice there is an increase of adenosine A1 receptors [41], and the different effect of TC‐2153 in neonatal vs adult mice could be due to the higher A1 receptor expression in the adult hippocampus. Thus, some of the effects of TC‐2153 could be due to the action of adenosine. Indeed, we found that TC‐2153‐induced STEP activation, an opposite effect with respect to its supposed activity as STEP inhibitor, was dependent on A1R stimulation. That the effect of STEP could depend on adenosine had already been demonstrated by our group in previous papers, where we identified a role for adenosine A2A receptor in the modulation of STEP activity, mainly through the interaction with metabotropic glutamate receptors type 5 [32, 42, 43]. Here we found that also adenosine A1 receptor can modulate STEP activity and even though we did not investigate the signaling pathway involved, it is known that A1 receptor is couple to G_i_ and to inhibition of Protein Kinase A (PKA) [44], and that inhibition of PKA promotes STEP activity [45, 46]. Therefore, we hypothesize that in hippocampal slices TC‐2153 exerts an initial action, STEP‐independent, that leads to adenosine release and consequently to STEP activation. When, instead, hippocampal slices are treated with TC‐2153 for longer periods, at least one hour, and in condition of STEP activation such as after LFS, TC‐2153 inhibits the tyrosine phosphatase. We did not investigate the mechanism through which TC‐2153 increased adenosine levels and induced A1R stimulation, but it is interesting to note that ketamine, a NMDA receptor antagonist that can rapidly produce antidepressant effects, may evoke a postsynaptic adenosine release, which retrogradely feedback onto A1R located on glutamatergic terminals and induce presynaptic inhibition of glutamate release [47]. Since TC‐2153 has antidepressant effects in mice [37] and, as ketamine, induces presynaptic A1R stimulation, it would be interesting to verify whether also TC‐2153 could have a rapid antidepressant effect in animal models.

Should the effect of TC‐2153 on adenosine release be confirmed also in vivo, the reported TC‐2153 effects in different models of brain damage [36, 48, 49] should be reconsidered in light of an involvement of adenosine. In fact, it is well known that adenosine exerts neuroprotective effects and modulates brain excitability. In particular, through A1R stimulation, adenosine exerts a clear anticonvulsive effect in different in vivo and in vitro models through the activation of K^+^ channels, which antagonizes membrane depolarization and renders neurons less excitable (see Spanoghe et al., 2020 for a review) [44]. Moreover, besides suppressing neuronal excitation through the opening of potassium channels, A1Rs inhibit Ca^2+^‐dependent presynaptic neurotransmitter release, further contributing to the reduction of excitation [50]. Through the same mechanisms, A1R stimulation exerts neuroprotective effects as shown in brain ischemia models [51, 52].

Thus, our data support the hypothesis that TC‐2153 rapidly induces adenosine release, triggering A1 receptor activation, which reduces synaptic transmission and activates STEP, potentially through PKA inhibition. These findings align with earlier reports demonstrating a role of adenosine in the regulation of STEP activity.

## 5 Conclusion

In conclusion, our work substantially enriches the understanding of STEP in hippocampal LTD and synaptic transmission. The unexpected, adenosine‐dependent activation of STEP by TC‐2153 represents a novel mechanism with significant experimental and therapeutic implications. Our results underscore the need for careful temporal analysis and mechanistic dissection when using pharmacological tools like TC‐2153 to modulate STEP activity.

## Author Contributions

V.C., R.P., and C.M. designed and performed the experiments; M.P., E.M., L.G., and Z.B. performed the experiments; V.C., R.P., C.M., and M.R.D. analyzed and interpreted the results; M.R.D. conceived and coordinated the project and wrote the manuscript, which was revised and commented on by all authors. All authors have read and agreed to the published version of the manuscript.

## Funding

This work was supported by Istituto Superiore di Sanità (Grant ISS‐FAR 23 and ISS‐FAR 24).

## Ethics Statement

All animal experiments met the European guidelines for the care and use of laboratory animals (2010/63/UE) and those of the Italian Ministry of Health (Decreto Legislativo 26/2014). The animal study protocol was approved by the Italian Ministry of Health (D9997.N.TNL) and by the local Institutional Animal Care and Use Committee (IACUC) at Istituto Superiore di Sanità (Rome, Italy).

## Consent

The authors have nothing to report.

## Conflicts of Interest

The authors declare no conflicts of interest.

## Data Availability

The data that support the findings of this study are available from the corresponding author upon reasonable request.
